# Considering social inequalities in health in large-scale testing for COVID-19 in Montréal: a qualitative case study

**DOI:** 10.1186/s12889-022-13163-3

**Published:** 2022-04-14

**Authors:** Marie-Catherine Gagnon-Dufresne, Lara Gautier, Camille Beaujoin, Ashley Savard Lamothe, Rachel Mikanagu, Patrick Cloos, Valéry Ridde, Kate Zinszer

**Affiliations:** 1 Department of social and preventive medicine, School of Public Health, University of Montréal, 7101 Park Avenue, Montréal, Québec H3N 1X9 Canada; 2 Centre de recherche en santé publique (CReSP), Montréal, Québec Canada; 3 Department of health management, evaluation and policy, School of Public Health, University of Montréal, Montréal, Québec Canada; 4 School of Social Work, University of Montréal, Montréal, Canada; 5 Centre Population et Développement, Institut de recherche pour le développement (IRD), Université de Paris, Paris, France

**Keywords:** COVID-19, SARS-CoV-2, Canada, Evaluation, Large-scale testing, Social inequalities in health, Equity, Health systems

## Abstract

**Background:**

Evidence continues to demonstrate that certain marginalised populations are disproportionately affected by COVID-19. While many studies document the impacts of COVID-19 on social inequalities in health, none has examined how public health responses to the pandemic have unfolded to address these inequities in Canada. The purpose of our study was to assess how social inequalities in health were considered in the design and planning of large-scale COVID-19 testing programs in Montréal (Québec, Canada).

**Methods:**

Part of the multicountry study HoSPiCOVID, this article reports on a qualitative case study of large-scale testing for COVID-19 in Montréal. We conducted semi-structured interviews with 19 stakeholders involved in planning large-scale testing or working with vulnerable populations during the pandemic. We developed interview guides and a codebook using existing literature on policy design and planning, and analysed data deductively and inductively using thematic analysis in NVivo.

**Results:**

Our findings suggest that large-scale COVID-19 testing in Montréal did not initially consider social inequalities in health in its design and planning phases. Considering the sense of urgency brought by the pandemic, participants noted the challenges linked to the uptake of an intersectoral approach and of a unified vision of social inequalities in health. However, adaptations were gradually made to large-scale testing to improve its accessibility, acceptability, and availability. Actors from the community sector, among others, played an important role in supporting the health sector to address the needs of specific subgroups of the population.

**Conclusions:**

These findings contribute to the reflections on the lessons learned from COVID-19, highlighting that public health programs must tackle structural barriers to accessing healthcare services during health crises. This will be necessary to ensure that pandemic preparedness and response, including large-scale testing, do not further increase social inequalities in health.

## Background

Evidence continues to accumulate highlighting that certain marginalised populations have been disproportionately affected by COVID-19 [1]. The pandemic has amplified existing health inequalities, shaped by long-standing structural inequities and socioeconomic determinants of health [2]. Public health systems and programs have a responsibility to protect the health of the population, including through emergency preparedness, paramount to ensuring that inequities are not exacerbated [3]. Central to infectious disease control and prevention is the ability to detect, monitor, and control disease transmission. Large-scale testing is instrumental in the case of infectious disease outbreaks, allowing for the detection of cases and resulting actions such as treatment, isolation, and contact tracing [4]. Inclusive and equitable access to testing programs is crucial to support response efforts [4, 5].

While many studies document the impacts of COVID-19 on social inequalities in health (SIH), to the best of our knowledge, none has examined how public health responses to the pandemic have unfolded to address these inequities in Canada. Research conducted elsewhere indicates that governments faced many challenges in developing testing strategies, such as the expansion of testing services for the whole population [6, 7]. Other studies demonstrate that large-scale testing for COVID-19 exemplified the hypothesised inverse care law, whereby those in greatest need are those who have the lowest access to interventions aimed at improving the health of the population [8, 9]. These findings from the current pandemic are important, as evidence shows that public health responses to infectious disease epidemics seldom consider SIH [4, 10].

Given this gap, we used Montréal as a case study to assess how SIH were considered in the design and planning of large-scale testing for COVID-19 [11]. Montréal, a city of approximately 2 million inhabitants located in the Canadian province of Québec, experienced one of the highest numbers of confirmed cases in the country during the first and second waves of the pandemic. The present article is based on the analysis of qualitative data from interviews with key informants involved in large-scale testing in Montréal. Understanding how large-scale testing was developed in Montréal and whether existing and emerging SIH were considered is not only critical for COVID-19, but also for future pandemics [4, 12].

## Methods

### Setting of the study

Canada’s healthcare system is largely publicly funded (70.9%), in which the federal government provides financial support to provinces and territories, who are responsible for organising and delivering healthcare services [13]. In the province of Québec, healthcare is under the responsibility of the Québec Ministry of Health and Social Services (MSSS) and includes public health. Public health represents approximately 2% of total health expenditures following recent budget cuts [14]. Territorially defined Integrated Centres for Health and Social Services (CISSS/CIUSSS, hereafter health centres) and public health departments are accountable to the MSSS. Montréal has one public health department and five health centres coordinating healthcare and social services to residents within their catchment area.

In Québec, COVID-19 was declared a public health emergency on March 13, 2020, soon followed by the implementation of SARS-CoV-2 diagnostic testing centres. As of May 2020, COVID-19 testing was open to Montréal residents with symptoms or without symptoms but having been in contact with confirmed cases [15]. During the first wave of COVID-19 (February 25 to July 11, 2020), public health departments were responsible for deciding on testing priorities, while health centres were responsible for designing and implementing large-scale testing. During the second (August 23, 2020 to March 20, 2021), third (March 21, 2021 to July 17, 2021), and subsequent waves, the responsibility for testing rested primarily with health centres, supported by public health departments [15]. Testing services varied between health centres, including permanent and mobile clinics, with and without appointments. COVID-19 testing, excluding testing required prior to or after international travel, has always been free for the population, with or without health insurance.

### Study design

This article is based on data from a descriptive qualitative case study [16] conducted in Montréal, which was part of the HoSPiCOVID study [11]. The case is the consideration of SIH in the design and planning of large-scale testing for COVID-19 in Montréal across space (different health centres responsible for planning large-scale testing in Montréal) and time (from the first wave of COVID-19 in March 2020 to the third wave in spring 2021). The case study describes large-scale testing interventions for the general population within their context, including their justification, scope, and the actors involved, as well as the adaptations over time.

### Sampling

Study participants were key informants directly or indirectly involved in the planning of large-scale testing for COVID-19 in Montréal, as well as other actors who worked with vulnerable populations during the pandemic. We identified a list of initial participants and used snowball sampling. We first recruited participants through the senior co-authors’ networks in Montréal. Participants then identified other actors that met the inclusion criteria. Inclusion criteria were as follows: (1) working in the health or community sector; (2) having participated in designing, planning, or implementing large-scale COVID-19 testing in Montréal; and (3) having been involved in the adaptation of large-scale testing over time or in creating parallel interventions targeting vulnerable populations. This strategy allowed the inclusion of not readily accessible individuals who could provide rich information on large-scale testing [17]. Thirty participants were initially identified and nineteen accepted to participate. The final sample was diverse and consisted of stakeholders from various settings [18].

### Data collection and characteristics of participants

The fourth and fifth authors conducted semi-structured interviews with participants between September 2020 and April 2021. Although data collection overlapped with the second and third waves of COVID-19 in Québec, interviews focused on the initial planning of large-scale testing during the first wave, and discussed adaptations made during the first, second, and third waves. Interviews were conducted in French via Zoom. They were audio recorded and transcribed in their original language. Excerpts used in study reports were subsequently translated into English by the first author, fluent in French and English. A total of 17 interviews, lasting between 30 minutes and 1 hour and a half, were conducted with 19 stakeholders.

The participants, 17 women and two men, came from various organisations and played different roles in large-scale testing (Table 1). Eleven participants worked in the health sector, and eight in community or philanthropic organisations. Four participants from the health sector were directly involved in developing large-scale testing clinics. Two participants from health centres worked to develop accessible communication strategies and intervention approaches to meet the needs of patients during COVID-19. Two others played strategic roles during COVID-19, ensuring that the health sector created partnerships and supported other sectors in adapting their activities and managing outbreaks in their respective environments. One participant had a position created specifically to tackle the pandemic, aimed at supporting actors from the community and health sectors in their responses to COVID-19. The remaining ten participants, three from the health sector and seven from the community sector, had different mandates directly related to vulnerable populations (*i.e.*, public health research and planning officer, project coordinator, researcher, community organiser, outreach worker), with nine specifically working with migrants and racialised minorities. One participant had positions both within the community and health sectors during the pandemic. While most participants were not directly involved in planning large-scale testing, their views as collaborators to health centres provided important information on the strengths and weaknesses of testing efforts in Montréal.

**Table 1:** Professional information on study participants (*N*=19)

Sector of employment	Role	Number of participants	Identification
Health sector (*n*=11)	Managerial position in a large-scale COVID-19 testing clinic	*n*=4	Participants 02, 04, 05, 09
	Support role for COVID-19 activities in a health centre (*i.e.*, community organiser, communication specialist, healthcare evaluation specialist, research coordinator)	*n*=4	Participants 08^a^, 10, 11, 16
	Public health position	*n*=3	Participants 03, 12, 13
Community and philanthropic sector (*n*=8)	Working with vulnerable populations in a community or philanthropic organisation (*i.e.*, director, project coordinator, community organiser, outreach worker)	*n*=7	Participants 01, 06, 14, 15, 17, 18, 19
	Specific role for COVID-19 in a community or philanthropic organisation	*n*=1	Participant 07

^a^Participant 08 occupied positions in the health and community sectors during the pandemic. Quotes were adjusted accordingly

### Theoretical bricolage

We created an interview guide and analysed data using a theoretical *bricolage* approach, a “do-it-yourself” strategy in which existing theories and frameworks are combined. Our *bricolage* was developed iteratively through multiple meetings with the HoSPiCOVID interdisciplinary research team. This approach was favoured based on previous work in the field of health policy and systems research, which typically involves scholars with diverse backgrounds and disciplinary traditions [19]. Qualitative researchers describe *bricolage* as the process of combining various elements (theories, concepts, tools) to address the complexity associated with certain social phenomena [20]. Public health scholars working on health inequalities have acknowledged the benefits associated with the process of mixing theories and conceptual frameworks, which can increase the value of health policy research [21]. Jones and colleagues (2021) indeed argue that, for those studying complex health systems, “multiple theories used together provide an overarching frame with more explanatory power for the policy processes in a given context” [19].

This methodological approach, sometimes referred to as “synthesis theories and frameworks” [22], reflects a broader trend in public policy research [23, 24] which, as health policy and systems scholars, we regularly engage with. For this case study examining intervention design and planning processes, we initially drew from public policy research (specifically, the seminal works by Howlett on the essential stages of policy design [25]) and Pineault’s major contribution, his book on public health planning [26]. However, we realised that neither of these authors’ works allowed for a comprehensive consideration of SIH. For this reason, we decided to include the REFLEX-ISS tool [27] in our conceptual reflections. The REFLEX-ISS tool was developed in Québec to assess the inclusion of SIH in planning public health interventions. These three strands of work allowed the development of a four-category *bricolage* framework. The combination process is detailed in Table 2. The first category refers to how respondents and their institutions perceived SIH, considering their understanding of the COVID-19 context and available evidence. The second describes the overarching strategy favoured by stakeholders to tackle SIH. It differentiates between location-based, vulnerability-based, and population-wide strategies. The third examines intersectoral collaboration and long-term involvement of multiple sectors. It specifically assesses how the community sector complemented efforts from the health sector to address SIH. The fourth refers to the adaptation capacity of large-scale testing, including the adoption of flexible design and planning with constant monitoring of emerging needs and evidence to mitigate unintended consequences. Adaptations include the improvement of accessibility (the opportunity to have healthcare needs fulfilled), acceptability (the sociocultural factors that determine the possibility for people to accept aspects of a healthcare service and its judged appropriateness), and availability (the capacity for people to reach healthcare services physically and in a timely manner) of testing services to address SIH, meeting the needs of specific populations [28].

**Table 2:**
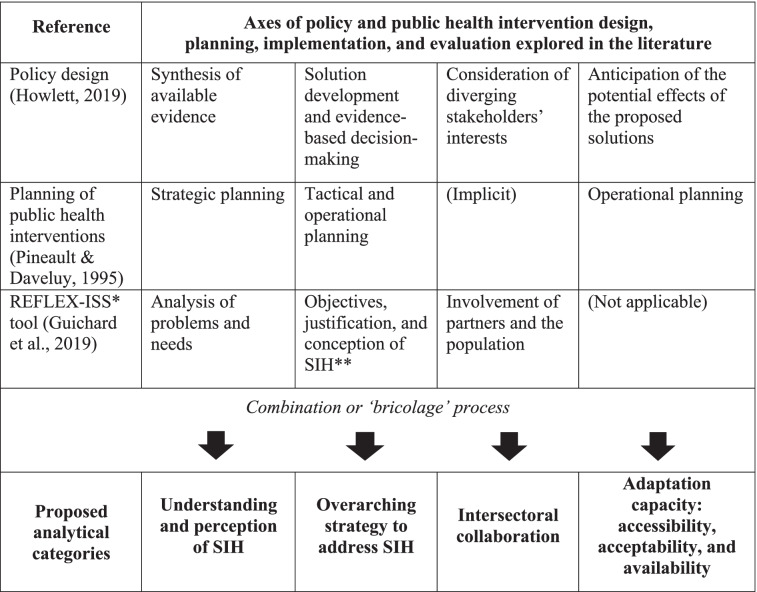
Theoretical *bricolage* developed for HoSPiCOVID

*REFLEX-ISS: Tool developed to assess the consideration of social inequalities in health in public health interventions. Its name comes from the French words *réflexions* (reflections) and *inégalités sociales de santé* (social inequalities in health)

**SIH: Social inequalities in health

### Data analysis

We analysed the interview data using thematic analysis [29]. The first author read the interview transcriptions multiple times and wrote memos to note initial code and theme ideas, as well as preliminary analytical insights. Data were analysed deductively and inductively in NVivo. Three initial transcripts were coded to create a preliminary list of codes, followed by the systematic coding of all transcripts [30]. As per an inductive coding approach, codes were developed inductively from the data to closely reflect the words of participants. As per a deductive coding approach, these codes were subsequently fitted into the four axes of the theoretical *bricolage* previously described [31]. Consequently, while codes and categories ascribed to interview excerpts were developed inductively, the themes used to regroup them were deductive, reflecting the content of the theoretical *bricolage* [31]. The codebook was adapted in an iterative manner throughout the coding process. Themes and their content were defined and reviewed with the research team.

### Reflexivity

All authors come from a public health background, with experience working with populations in situations of vulnerability in Canada and abroad. We all believe that public health interventions should better consider SIH. We nevertheless acknowledge that planning public health interventions in times of crisis is challenging, especially in a context where resources allocated for public health are scarce. We come from diverse backgrounds and sectors, and our views as academics might differ from those of the participants interviewed, who are professionals working in the health and community sectors. We recognise that our postures and experiences may have influenced the data collection process and our interpretation of findings.

### Ethical considerations

Ethical approval was granted by the Science and Health Research Ethics Board at the University of Montréal for the entire project (CERSES-20-061-D). All participants interviewed were informed about the aim of the study beforehand, consented to participate in the study, and gave their written informed consent. All analyses were performed on de-identified data. The protocol for involving human data was in accordance with the guidelines of the Canadian Tri-Council Policy Statement 2 on Ethical Conduct for Research Involving Humans (TCPS-2) and the Declaration of Helsinki.

## Results

### Different professional affiliations, different visions: Understanding and perception of SIH

Diverse perceptions of SIH emerged from our analysis of interview data. Most respondents mentioned that SIH were an important part of their organisation’s vision and mandate, both before and during the pandemic. Participants from different organisations had different perceptions of SIH, which seemed to be related to the role played by their respective organisation in managing the pandemic:

We are always confronted with SIH. […] One of my mandates is ensuring that we meet the needs of the whole population. We know that our health centre is situated [in a low-income neighbourhood] and that our target population faces health and social deprivation. […] In the context of my current mandate, I always deal with SIH in making decisions. (Participant 09, health sector)

[I think the objectives of large-scale testing] only partly consider SIH, not fully… We’ve been in the field for months and […] if I come out of my “community sector bubble,” I don’t really see changes. […] I didn’t see initiatives put in place, except by the community sector […] I don’t think that large-scale testing decreases inequalities, because there are no specific services [for people with difficult living conditions]. (Participant 06, community sector)

Another participant also emphasised these different visions of SIH across different levels within the health sector:

You were asking me earlier if there was a common philosophy or perception of SIH… There is no doubt that with our colleagues in Montréal, we perceive SIH in a specific way, but at the level of the Ministry of Health more generally, or even of public health departments, it can really differ. (Participant 16, health sector)

Some participants perceived that the fact that sociodemographic data on COVID-19 cases were not collected by the Québec government reflected this generalised lack of consideration for SIH:

The issue is that people who experience social inequalities are also those who are disproportionately impacted by the pandemic […] It’s extremely frustrating in Québec that we don’t have access to data on racialised populations, income, language… on all these elements that are important determinants for the pandemic, as we saw in Ontario and other countries. (Participant 08, health sector)

Most participants had some knowledge of SIH. However, testimonies from participants suggest that there was not a clear and unified vision of SIH among actors and organisations involved in planning large-scale testing in Montréal, either between the health and community sectors (Participants 09 and 06); within the health sector (Participants 16); or between the health sector and the government (Participant 08). For instance, while some understood SIH as closely related to equality (giving the same treatment to everyone), others viewed SIH through an equity lens (giving more to those in greatest need). In turn, these heterogeneous perceptions of SIH and the lack of data on the groups most affected by COVID-19 in Montréal seemed to have hindered efforts at considering SIH in planning large-scale testing.

### Between meeting the needs of the whole population and those of vulnerable subgroups: Overarching strategy to address SIH

#### State of emergency and lack of design

Many respondents mentioned that the state of emergency caused by the pandemic was unprecedented. This impeded the adequate and evidence-based design and planning of large-scale COVID-19 testing, as well as the consideration of SIH given the immediacy of the situation. For instance, two participants who contributed to planning testing clinics stated that their health centre did not have a well-developed protocol for such interventions:

We really started from zero. […] I’m trying to think about what we had in terms of screening services [in our hospital], and nothing comes to mind. […] So, of course, it was a lot of “trial and error” in the end. (Participant 04, health sector)

Sometimes, before COVID, we discussed health crises, and I think it was an abstract concept. We thought that it was possible to plan in a crisis context… But in a real crisis, planning is very short term. (Participant 09, health sector)

Another participant who managed a COVID-19 testing clinic emphasised that the priority during the first wave of COVID-19 was to develop large-scale testing as quickly as possible, without considering SIH, because they did not have the initial resources and tools in place. She mentioned that this lack of design was not necessarily undesirable:

I wonder if we would have been able to think about social inequalities, you know… At the same time as organising this clinic and… I don’t know. I’m under the impression that it’s maybe not a bad thing to work towards responding positively to emerging needs. (Participant 02)

Participants highlighted that there was a lack of pre-existing guidelines to design large-scale testing for COVID-19, and that initiatives were not always grounded in lessons learned from health crises in other contexts. The state of emergency brought by the pandemic thus hindered the use of evidence-based design and planning.

#### Primacy of a population-wide strategy for the health sector

In line with the visions of SIH described early, participants from the health sector mentioned that large-scale testing was first designed as a universal intervention, planned to reach the whole population. A community organiser mentioned that “the objectives [of large-scale testing] are to make testing accessible to everyone” (Participant 08, health sector). Similarly, respondents involved in developing large-scale testing explained that employing a population-wide strategy first and subsequently addressing SIH represented a normal course of actions:

When we opened [our first testing centre], the message that we were putting forward was “come and get tested, we want you to get tested.” So, men, women, everyone… [Testing was available] for everyone. And then, we realised with our team […] that our service offer wasn’t working [for specific populations]. (Participant 02)

When you plan something for the masses… You can’t think of social inequalities in health. […] You’re creating a new service for the whole population, so you must create it – at the beginning, I mean at the very beginning only – by not thinking about that. But once the foundations are there, then you can start to think about, “OK, with what I have, am I able to reach everyone? What is the population I can’t reach? How can I adapt this universal service?” (Participant 09)

Participants thus perceived that large-scale testing was designed and implemented for the whole population first, in the hopes of being reactive and increasing access to screening services for everyone. This suggests that they prioritised a phased strategy, as interventions to tackle potential SIH only came after the population-wide testing services were implemented.

### Heterogeneous planning and dependence on the health sector: Intersectoral collaboration

#### Shifting of the decision-making centre

Most participants recognised that the health sector was responsible for designing, planning, and implementing COVID-19 testing programs in Montréal. Some respondents mentioned that between the first and subsequent waves of COVID-19, there was a shift in decision-making and coordination from the regional level, with public health departments and the Ministry of Health and Social Services, to territorially defined health centres:

We created partnerships for large-scale testing. At one point, we had [services planned for] the whole Island of Montréal, and then health centres took over. We were in partnerships with other health centres, because to tackle inequalities in accessing health services […] we should offer services where populations are located. So, the Ministry of Health decided – I think it was during the summer – that health centres would take over large-scale testing [to offer services to populations in their catchment area]. (Participant 05, health sector)

Accordingly, some participants recognised health centres as the “main actors” in large-scale testing, stressing that “the planning [of large-scale testing] was very different between each health centre” (Participant 13, public health). Participants from the community sector mentioned that even if they participated in working groups, they were unsatisfied because of their lack of autonomy and decision-making power:

[Working groups] don’t have the money. [*Ad hoc*] committees don’t have the money. Health centres can give us human resources and money. They go with what they can [offer] […] In terms of an action plan, we try to adapt our actions to the reality of health centres. (Participant 01, community sector)

The responsibility for executive decision-making thus evolved during the pandemic in Montréal, shifting from the regional level to local health centres, with varying involvement from other actors depending on where the decision-making power rested.

#### Attempts at formalising an intersectoral approach to large-scale testing

While the level of collaboration with partners varied between each health centre, many participants from the health sector stated that the pandemic increased the collaboration between different stakeholders: “A positive aspect of the pandemic is that it very clearly reinforced the collaboration between public health and the community and research sectors” (Participant 16). Collaboration between sectors was formalised through the creation of crisis units and working groups, involving hospitals and public health departments, decision makers at the city and neighbourhood levels, and community organisations, among others:

From the first wave, we created what we called crisis units, in collaboration with city districts and community partners. These units brought together hospitals, districts, […] neighbourhood groups and key community partners to try and develop constant consultations to be able to intervene quickly and share information on upcoming large-scale testing clinics. (Participant 12, public health)

This collaboration largely centred around the health sector, consulting and sharing information to support other organisations working with communities. However, some community actors felt that their involvement in large-scale testing was minimal, and that they did not receive enough support from the government to appropriately care for communities:

[The government] gives us information and, “figure it out with the information that we give you” and that’s it. […] [COVID-19] started in March, and I think it’s only in September that we received emergency funds. […] There’s a clear delay compared to services offered to the general population. (Participant 17, community sector)

And the exhaustion to always try to address these issues while it shouldn’t be the community sector’s responsibility. […] We shouldn’t be the people on the front lines… It should be hospitals, public health departments, the government, local authorities that come and really address [SIH]. (Participant 06, community sector)

Therefore, the health sector was the key stakeholder involved in large-scale testing, collaborating with other actors to share governmental guidelines and support partners. Yet, data suggests that resources were insufficient to address SIH.

### Gradual joint efforts to reach vulnerable groups: Adaptation capacity of large-scale testing

#### Accessibility of large-scale testing

Various adaptations were made to large-scale testing to increase accessibility for specific population groups, addressing information, linguistic, physical, and geographic obstacles to COVID-19 testing. Access to information was a barrier identified by several participants. Accordingly, the adaptation that was most discussed was the written and oral translation of testing-related information in multiple languages to better reach allophone communities:

Another problem with planning was information on testing. […] This must be communicated very broadly to the population, through various means and in different languages, which wasn’t done at the beginning. We worked with a [community organisation] offering translation services […] which later contributed to translating material for other territories, allowing health centres to translate their material in various languages. (Participant 07, philanthropic sector)

Some participants also mentioned that information about large-scale testing was adapted for low literacy levels through collaborations between the health and community sectors:

We have done another series [of informative materials] that used pictograms, very few words and pictograms. […] We sometimes had templates that had too much text, so we went by trial-and-error… But it was something we thought about. (Participant 12, health sector)

Other adaptations discussed were the development of outpatient testing and partnerships with taxi companies to increase access to testing for individuals with mobility issues or low-income, and for the elderly:

We were asked to go and test people at home […] Our home-based services aimed at reaching people in long-term care facilities, private retirement homes, rehabilitation centres for people with intellectual disabilities, youth shelters […] This really allowed us to target a vulnerable clientele. (Participant 02, health sector)

We looked if it was possible to provide taxi fares for people who could not [get tested]. We had an agreement with [a taxi company] so that people at risk who couldn’t come could get transport. (Participant 05, health sector)

Many adaptations to COVID-19 testing occurring after the initial implementation were mentioned by participants, which included improving linguistic, informational, physical, and geographic accessibility.

#### Acceptability of COVID-19 testing

Efforts were made in collaboration with partners outside the health sector to improve the acceptability of COVID-19 testing for certain population groups. One of the major obstacles discussed by respondents was the difficulty of some people to comply with self-isolation recommendations because of their vulnerable social positions in case they tested positive to COVID-19, influencing their initial decision to get tested:

Sometimes, there are occupational health teams present in the field in the case of outbreaks to inform people […] about their rights for the 14 days [of self-isolation]. […] There are also discussions with the Red Cross to provide care packages during self-isolation. […] There are discussions with foundations to see […] how we could find money to pay these people so they can isolate. It’s a public health mandate and many actions are taken towards this goal. (Participant 03, public health)

One participant also mentioned that additional efforts were made to encourage homeless individuals to get tested and follow governmental sanitary guidelines:

Obviously, patients experiencing homelessness needed a place to wait for their results, and needed a place to self-isolate, because most of the community organisations were not welcoming them anymore. So, we got that going. (Participant 02, health sector)

Some community groups also supported the right of migrants without medical insurance to be tested for COVID-19 without fear of being reported or getting into trouble with authorities:

Some groups highlighted the issue of having a [health insurance card]. It was written on posters that you needed a health insurance card to get tested. So, there were many efforts to say, “We can’t put that on posters because people without an [authorised migrant] status, who don’t have the card, won’t want to get tested.” […] This can be an example of how we listened to what came from the field to adapt practices. (Participant 08, health sector)

Consequently, adaptations were made to large-scale testing to increase its acceptability for different vulnerable groups. Many of these changes were motivated by pressures from the community sector, advocating to remove barriers influencing how various groups perceived large-scale testing.

#### Availability of services

Adaptations to large-scale testing also aimed at improving the physical and timely availability of services, specifically targeted at reaching vulnerable populations in their environment and at broadening the offer of services for them. These adaptations unfolded in three specific ways. First, most participants mentioned that the creation of mobile clinics in collaboration with community actors improved service provision for vulnerable groups:

We had regular clinics covering our territory, and we added mobile clinics too, to move closer to red zones with populations that we thought would not necessarily go [to regular clinics]. […] We worked in partnership with community organisations to promote and organise clinics. (Participant 12, public health)

Second, the transition from appointment only to walk-in clinics was mentioned by many as an adaptation that increased the availability of testing services:

Services were [initially] very focused on appointment booking. We believed, particularly in certain communities, that it was easier in terms of people’s working conditions or daily life to go to walk-in clinics. This offer is increasing with time… But we should have been more flexible from the beginning and create walk-in clinics to accommodate as many people as possible. (Participant 07, philanthropic sector)

Third, the schedules of testing clinics were modified to offer longer operating hours:

We had to think about […] which operating hours could best tackle issues [of service availability]. […] We were suggesting that evenings were better, because a lot of people in the field were telling me they couldn’t go during the day. It took a long time for requests to be heard. […] Now it’s better in terms of location, operating hours, and advertising. (Participant 08, community sector)

Several adaptations were thus implemented to improve the availability of testing services in Montréal, improving the offer of services for vulnerable populations. While participants mentioned that it was difficult to change existing interventions, they recognised that services improved gradually to respond to the needs of specific subgroups. Nevertheless, most participants highlighted that these changes should have come sooner and that there was still work to do to ensure that testing programs reached vulnerable and at-risk groups in Montréal.

## Discussion

This qualitative study provided useful insights on the consideration of SIH in the design and planning of large-scale testing for COVID-19 in Montréal (Canada). The results suggest that there was not a common vision of SIH among actors involved in large-scale testing in Montréal. In addition, the unprecedented scale and speed of the pandemic in combination with a lack of pre-existing guidelines for emergency preparedness led to a population-wide strategy being prioritised for COVID-19 testing. The organisation of the response to COVID-19 in Montréal, largely centred around the health sector, represented an important challenge for the uptake of intersectoral collaboration with other sectors. However, various adaptations were gradually made to increase the accessibility, acceptability, and availability of testing programs. The community sector played an important supporting role in these adaptations. This study is significant as, to the best of our knowledge, it is one of the first to investigate the consideration of SIH in testing efforts. Our findings can be used to improve current and future testing initiatives in the context of infectious disease outbreaks.

Our theoretical *bricolage,* focused primarily on SIH, was innovative and allowed to identify the interactions between various levels of governance and activities for large-scale testing, including their adaptations. Combining existing theories and conceptual frameworks to fit research objectives is a promising approach to health policy research, as it provides a flexible and holistic tool for understanding complex interventions in a given context [19].

Our first theme demonstrated that there lacked a unified vision of SIH for all sectors involved in large-scale testing in Montréal. It has been argued elsewhere that the ways in which different actors understand and represent SIH (perception of SIH), their causes and their consequences influence how health interventions are designed (strategy to tackle SIH) [32]. In our study, it seems contradictory that many participants recognised the need to reach vulnerable groups not to exacerbate inequities, but that the overarching strategy to large-scale testing was population-wide (as demonstrated by our second theme). This strategy, aimed at giving the same treatment to everyone, is, however, centred on a vision of equality rather than equity [33]. In addition, participants shared their frustration towards the fact that the Québec government did not collect disaggregated data on COVID-19 cases, which would have been useful for evidence-based decision-making for large-scale testing. Indeed, recent studies highlighted the importance of collecting data on social determinants of health (*i.e.*, gender, age, occupation, income, ethnicity) to support decision-making during COVID-19, considering that disadvantaged social contexts were associated with increased risk of infection [34, 35]. Adopting an integrated vision of SIH for all stakeholders involved in developing public health measures such as large-scale testing could have played a key role in identifying the most appropriate strategies for reaching specific targeted groups [36].

The second theme showed that the state of urgency created by COVID-19 resulted in large-scale testing being developed without consulting or using evidence from prior infectious disease outbreaks. Other studies have demonstrated that governments worldwide failed to adopt evidence-based decision-making during COVID-19, notably for planning large-scale testing and tackling SIH [37, 38]. This echoes the findings of recent rapid reviews, emphasising that health inequity is rarely considered in the design and evaluation of public health interventions [4, 10]. Evidence shows that population-wide strategies tend to exacerbate SIH [39, 40]. Benefits from population-wide strategies are not equally distributed across a population, as they often fail to address the social conditions disadvantaging certain groups [39, 40]. In contrast, strategies centred around proportionate universalism – whereby services are available for the whole populations but the intensity of the efforts is adapted to the level of disadvantage of various population groups – are more promising, as they target the conditions that prevent some populations from using public health resources to improve their health [39]. This type of strategy could have contributed to better considering existing SIH, while also responding to the newly emerging vulnerabilities for some population groups because of the pandemic (*i.e.*, the elderly in long-term care facilities, essential service workers). A population-wide strategy was nevertheless the initial overarching strategy to large-scale testing adopted in Montréal, despite the Canadian Pandemic Influenza Preparedness plan indicating that planners must identify specific populations, settings, and needs for prevention or care services [41]. Notwithstanding the challenges associated with planning public health initiatives in sanitary crises, the uptake of an evidence-based strategy to address SIH in large-scale testing could have allowed a better consideration of SIH in Montréal.

The third theme highlighted that decision-making for testing was primarily with the health sector. Accordingly, participants from the community sector perceived that their involvement was minimal and that they did not have enough resources to tackle SIH, despite being front line workers throughout the pandemic. Other studies suggest that integrated and inclusive governance for health is crucial to tackle COVID-19 [42]. They emphasised the importance of multilevel and multisectoral approaches, coupled with community participation and collaboration with community organisations, to promote social protections and foster equity [42, 43]. The creation of mutually beneficial partnerships with the community sector and the population is presented as integral part of the public health apparatus in Québec, despite interventions seldom being adapted to their local context and involving community actors in practice [44, 45]. This could have been improved in the context of COVID-19, as collaboration appears to be critical for reaching vulnerable populations in health crises [46].

Our fourth theme indicated that large-scale testing was iteratively adapted to increase the accessibility, acceptability, and availability of services. Recent studies suggest that inability to conduct physical distancing, precarious working conditions, and limited access to accurate information were potential reasons for the unequal uptake of testing during COVID-19 [47, 48]. Adaptations that were made in Montréal to improve the reach of testing services include translating and simplifying testing-related information, testing people in their homes, organising mobile clinics in at-risk neighbourhoods, providing support for people self-isolating, and widening operating hours of testing centres. Other recent studies discuss adaptations to testing programs that were implemented elsewhere. Mobile clinics have been deployed in low-income neighbourhoods in partnership with community organisations in the United States [49–51], and have been coupled with home visits in Italy [52]. Measures to support people undergoing self-isolation (*i.e.*, financial aid, food and other supplies) have been set by governments in Singapore, Japan, China, South Korea, the United States and the UK [53]. Translation of information related to COVID-19 was done for migrant populations in various countries, such as Turkey, Qatar and the United States [54–56]. While COVID-19 materials related to disease education and behaviour change were translated in most European countries, specific information on testing procedures and entitlements to healthcare services during the pandemic were only translated in three of them (the UK, Denmark, and the Netherlands) [57, 58].

Reinvention of public health interventions as they are implemented can improve their effectiveness, showing that they are flexible enough to be adapted to emerging local needs [59]. Targeted outreach efforts in community settings, involvement of community leaders and organisations, and cultural adaptation of services were identified as promising strategies for increasing the accessibility, acceptability, and availability of large-scale testing [48, 60]. Adapting testing strategies to contextual factors in collaboration with community actors and organisations, which was gradually done in Montréal, was therefore an important step in mitigating the negative impacts of COVID-19 policies on SIH [61].

### Limitations

First, due to the strain caused by COVID-19 on healthcare systems and their personnel, some actors involved in planning large-scale testing could not be reached or did not accept our invitation to participate. Accordingly, our study only included health sector participants from two territories, despite there being five in Montréal. This resulted in the sample not necessarily representing the experiences of all testing sites. Second, while our study focused on the design and planning of large-scale testing, many respondents were only indirectly involved in this phase and thus mainly discussed implementation. The inclusion of key informants with various affiliations nevertheless offered a nuanced and comprehensive view of testing programs, and the use of thick descriptions and direct quotes increased the transferability of findings [62]. Third, interview excerpts were translated into English for reporting purposes. While quotes were validated multiple times, translation could influence the comparability of data, reflecting choices made by the translator about form and content [63]. Fourth, although this could have increased the credibility of findings [62], transcripts were not returned to participants for member checking as we did not want to further increase their workload considering their role in managing the pandemic. However, knowledge transfer activities will be organised to discuss findings with knowledge users, including participants and other actors involved in large-scale testing for COVID-19 in Montréal. Lessons learned will be identified with knowledge users and policy briefs will be created for decision makers, to improve the response to future epidemics. Fifth, our theoretical *bricolage* represented an attempt at classifying interview data, and themes were not mutually exclusive. This *bricolage* was nonetheless useful in classifying and analysing data, ensuring that results were presented in a coherent manner.

## Conclusions

Despite repeated calls for public health to improve social justice, our study suggests that SIH were initially not prioritised in large-scale testing in Montréal [64, 65]. From the Ottawa Charter [65] to the Commission on the Social Determinants of Health [39], COVID-19 shows that we must continue to advocate for SIH to be central in public health initiatives. Pandemic preparedness and response must include a commitment to truly “leave no one behind.” This will not happen without political will and a substantial increase in the resources available for public health and the reduction of SIH, in Québec and elsewhere.

## Data Availability

The data analysed during the current study are not publicly available due to confidentiality concerns for participants. Deidentified transcripts of the interviews are available from the corresponding author upon reasonable request.

## Abbreviations

SIH: Social Inequalities in Health
MSSS: Québec Ministry of Health and Social Services
CISSS/CIUSSS: Integrated (University) Centre for Health and Social Services

## References

[1] Upshaw TL, Brown C, Smith R, Perri M, Ziegler C, Pinto AD. Social determinants of COVID-19 incidence and outcomes: A rapid review. PLoS ONE [Internet]. 2021;16(3). [cited 2021 Aug 30]. Available from: https://journals.plos.org/plosone/article?id=10.1371/journal.pone.0248336. 10.1371/journal.pone.0248336 PMC8011781 33788848

[2] Greenaway C, Hargreaves S, Barkati S, Coyle CM, Gobbi F, Veizis A (2020). COVID-19: Exposing and addressing health disparities among ethnic minorities and migrants. J Travel Med..

[3] Benjamin GC. Ensuring health equity during the COVID-19 pandemic: the role of public health infrastructure. Rev Panam Salud Pública. 2020:1–4. 10.26633/RPSP.2020.70 PMC7279120 32523608

[4] Ost K, Duquesne L, Duguay C, Traverson L, Mathevet I, Ridde V, et al. A rapid review of equity considerations in large-scale testing campaigns during infectious disease epidemics. Preprint [Internet]. 2021. [cited 2022 Mar 02]. Available from: https://www.medrxiv.org/content/10.1101/2021.02.22.21252205v2.full.pdf.

[5] Berger ZD, Evans NG, Phelan AL, Silverman RD (2020). Covid-19: control measures must be equitable and inclusive. BMJ..

[6] Magno L, Rossi TA, Mendonça-Lima FW, Dos Santos CC, Campos GB, Marques LM (2020). Challenges and proposals for scaling up COVID-19 testing and diagnosis in Brazil. Ciênc Saúde Coletiva..

[7] Thébaud-Mony A (2020). Lessons From Tuberculosis Control for COVID-19: Screening Should Be Universal. NEW Solut J Environ Occup Health Policy..

[8] Green MA, García-Fiñana M, Barr B, Burnside G, Cheyne CP, Hughes D (2021). Evaluating social and spatial inequalities of large scale rapid lateral flow SARS-CoV-2 antigen testing in COVID-19 management: An observational study of Liverpool, UK (November 2020 to January 2021). Lancet Reg Health Eur..

[9] Riou J, Panczak R, Althaus C, Junker C, Perisa D, Schneider K, et al. From testing to mortality: COVID-19 and the inverse care law in Switzerland. Top Antivir Med. 2021:40–0. 10.1016/S2468-2667(21)00160-2 PMC8270761 34252364

[10] Mathevet I, Ost K, Traverson L, Zinszer K, Ridde V (2021). Accounting for health inequities in the design of contact tracing interventions: A rapid review. Int J Infect Dis..

[11] Ridde V, Gautier L, Dagenais C, Chabrol F, Hou R, Bonnet E (2021). Learning from public health and hospital resilience to the SARS-CoV-2 pandemic: protocol for a multiple case study (Brazil, Canada, China, France, Japan, and Mali). Health Res Policy Syst..

[12] Araz OM, Ramirez-Nafarrate A, Jehn M, Wilson FA (2020). The importance of widespread testing for COVID-19 pandemic: systems thinking for drive-through testing sites. Health Syst..

[13] Government of Canada. Canada’s health care system [Internet]. Government of Canada. 2016. [cited 2021 Aug 23]. Available from: https://www.canada.ca/en/health-canada/services/canada-health-care-system.html.

[14] Denis J-L, Potvin L, Rochon J, Fournier P, Gauvin L (2020). On redesigning public health in Québec: lessons learned from the pandemic. Can J Public Health..

[15] Institut national de santé publique du Québec. Ligne du temps COVID-19 au Québec [Internet]. Institut national de santé publique du Québec (INSPQ). 2021. [cited 2021 Jul 23]. Available from: https://www.inspq.qc.ca/covid-19/donnees/ligne-du-temps.

[16] Merriam SB, Tisdell EJ. Qualitative Research: A Guide to Design and Implementation. 4th ed. San Francisco (CA): John Wiley & Sons; 2016.

[17] Palinkas LA, Horwitz SM, Green CA, Wisdom JP, Duan N, Hoagwood K (2015). Purposeful sampling for qualitative data collection and analysis in mixed method implementation research. Adm Policy Ment Health..

[18] Pires A, Poupart D, Groulz L, Mayer, Pires (1997). Échantillonnage et recherche qualitative: essai théorique et méthodologique. La recherche qualitative: Enjeux épistémologiques et méthodologiques.

[19] Jones CM, Gautier L, Ridde V (2021). A scoping review of theories and conceptual frameworks used to analyse health financing policy processes in sub-Saharan Africa. Health Policy Plan..

[20] Denzin NK, Lincoln YS (2011). The SAGE Handbook of Qualitative Research.

[21] Fran B (2018). People’s health and the social determinants of health. Health Promot J Austr..

[22] Nowlin MC (2011). Theories of the policy process: State of the research and emerging trends. Policy Stud J..

[23] Pierce JJ, Peterson HL, Jones MD, Garrard SP, Vu T (2017). There and Back Again: A Tale of the Advocacy Coalition Framework. Policy Stud J..

[24] Jones MD, Peterson HL, Pierce JJ, Herweg N, Bernal A, Lamberta Raney H (2016). A River Runs Through It: A Multiple Streams Meta-Review. Policy Stud J..

[25] Howlett M (2019). Designing Public Policies: Principles and Instruments.

[26] Pineault R, Daveluy C (1995). La Planification de la santé: concepts, méthodes, stratégies.

[27] Guichard A, Tardieu É, Nour K, Lafontaine G, Ridde V (2019). Adapting a health equity tool to meet professional needs (Québec, Canada). Health Promot Int..

[28] Levesque J-F, Harris MF, Russell G (2013). Patient-centred access to health care: conceptualising access at the interface of health systems and populations. Int J Equity Health..

[29] Braun V, Clarke V (2006). Using thematic analysis in psychology. Qual Res Psychol..

[30] Ritchie J, Spencer L, Brymand A, Burgess RG (1994). Qualitative data analysis for applied policy research (1st edition). Analyzing qualitative data.

[31] Saldaña J (2013). The coding manual for qualitative researchers.

[32] Ridde V, Guichard A. Réduire les inégalités sociales de santé: aporie, épistémologie et défis [Internet]. Presses de l’EHESP; 2008. [cited 2021 Aug 24]. Available from: https://www.cairn.info/lutter-contre-les-inegalites-sociales-de-sante%2D%2D9782859529840-page-57.htm.

[33] Potvin L, Ridde V, Mantoura P. Évaluer l’équité en promotion de la santé. In: Bernard P, Demers A, Frohlich K, De Koninck M, editors. Les inégalités sociales de santé au Québec [Internet]. Montréal: Presses de l’Université de Montréal; 2018. p. 355–78. [cited 2022 Mar 2]. (Paramètres). Available from: http://books.openedition.org/pum/10025.

[34] Yaya S, Yeboah H, Charles CH, Otu A, Labonte R (2020). Ethnic and racial disparities in COVID-19-related deaths: counting the trees, hiding the forest. BMJ Glob Health..

[35] Khalatbari-Soltani S, Cumming RG, Delpierre C, Kelly-Irving M. Importance of collecting data on socioeconomic determinants from the early stage of the COVID-19 outbreak onwards. J Epidemiol Community Health. 2020; jech-2020-214297. 10.1136/jech-2020-214297 PMC7298202 32385126

[36] Public Health Agency of Canada. From Risk to Resilience: An Equity Approach to COVID-19 [Internet]. Canada: Public Health Agency of Canada; 2020. [cited 2021 May 31]. Available from: https://www.canada.ca/content/dam/phac-aspc/documents/corporate/publications/chief-public-health-officer-reports-state-public-health-canada/from-risk-resilience-equity-approach-covid-19/cpho-covid-report-eng.pdf.

[37] Moatti J-P (2020). The French response to COVID-19: intrinsic difficulties at the interface of science, public health, and policy. Lancet Public Health..

[38] Solinas-Saunders M, The US (2020). Federal Response to COVID-19 During the First 3 Months of the Outbreak: Was an Evidence-Based Approach an Option?. Am Rev Public Adm..

[39] Marmot M, Bell R (2012). Fair society, healthy lives. Public Health..

[40] Frohlich KL, Potvin L (2008). Transcending the Known in Public Health Practice - The Inequality Paradox: The Population Approach and Vulnerable Populations. Am J Public Health..

[41] Public Health Agency of Canada. Canadian Pandemic Influenza Preparedness: Planning Guidance for the Health Sector [Internet]. Government of Canada. 2018. [cited 2021 Jul 26]. Available from: https://www.canada.ca/en/public-health/services/flu-influenza/canadian-pandemic-influenza-preparedness-planning-guidance-health-sector/table-of-contents.html.

[42] Rajan D, Koch K, Rohrer K, Bajnoczki C, Socha A, Voss M (2020). Governance of the Covid-19 response: a call for more inclusive and transparent decision-making. BMJ Glob Health..

[43] Lal A, Erondu NA, Heymann DL, Gitahi G, Yates R (2021). Fragmented health systems in COVID-19: rectifying the misalignment between global health security and universal health coverage. Lancet..

[44] Ridde V, Druetz T (2016). La disparition de la communauté en santé publique et santé mondiale : origine sémantique, pragmatique ou contextuelle.

[45] Touati N, Garakani T, Charest É, Proteau-Dupont É. Des personnes uniques avant tout : une grille d’analyse critique pour mieux prendre en compte la diversité des besoins dans le cadre des actions sur les déterminants sociaux de la santé. Éthique Publique Rev Int D’éthique Sociétale Gouv. 2018;20(2).

[46] Gautier L. A year on – how community-based workers have strived to provide continuous support to vulnerable migrant populations, in Montreal and around the globe [Internet]. IHP: International Health Policies. 2021. [cited 2021 Aug 24]. Available from: https://www.internationalhealthpolicies.org/featured-article/a-year-on-how-community-based-workers-have-strived-to-provide-continuous-support-to-vulnerable-migrant-populations-in-montreal-and-around-the-globe/.

[47] Dodds C, Fakoya I (2020). Covid-19: ensuring equality of access to testing for ethnic minorities. BMJ..

[48] Valeriani G, Sarajlic Vukovic I, Lindegaard T, Felizia R, Mollica R, Andersson G (2020). Addressing Healthcare Gaps in Sweden during the COVID-19 Outbreak: On Community Outreach and Empowering Ethnic Minority Groups in a Digitalized Context. Healthcare..

[49] Kim HN, Lan KF, Nkyekyer E, Neme S, Pierre-Louis M, Chew L (2020). Assessment of Disparities in COVID-19 Testing and Infection Across Language Groups in Seattle, Washington. JAMA Netw Open..

[50] Attipoe-Dorcoo S, Delgado R, Gupta A, Bennet J, Oriol NE, Jain SH (2020). Mobile health clinic model in the COVID-19 pandemic: lessons learned and opportunities for policy changes and innovation. Int J Equity Health..

[51] Baker DR, Cadet K, Mani S. COVID-19 Testing and Social Determinants of Health Among Disadvantaged Baltimore Neighborhoods: A Community Mobile Health Clinic Outreach Model. Popul Health Manag [Internet]. 2021;24(6). [cited 2022 Feb 21]. Available from: https://www.liebertpub.com/doi/abs/10.1089/pop.2021.0066. 10.1089/pop.2021.0066 PMC8851210 34030489

[52] Nacoti M, Ciocca A, Brambillasca P, Fazzi F, Pisano M, Giupponi M, et al. A Community-Based Model to the COVID-19 Humanitarian Crisis. Front Cell Infect Microbiol [Internet]. 2021;11. [cited 2022 Feb 21]. Available from: https://www.frontiersin.org/article/10.3389/fcimb.2021.639579. 10.3389/fcimb.2021.639579 PMC8009176 33796484

[53] Chung S-C, Marlow S, Tobias N, Alogna A, Alogna I, You S-L (2021). Lessons from countries implementing find, test, trace, isolation and support policies in the rapid response of the COVID-19 pandemic: a systematic review. BMJ Open..

[54] Bahar Özvarış Ş, Kayı İ, Mardin D, Sakarya S, Ekzayez A, Meagher K (2020). COVID-19 barriers and response strategies for refugees and undocumented migrants in Turkey. J Migr Health..

[55] Ahmad R, Hillman S (2021). Laboring to communicate: Use of migrant languages in COVID-19 awareness campaign in Qatar. Multilingua..

[56] Behbahani S, Smith CA, Carvalho M, Warren CJ, Gregory M, Silva NA. Vulnerable Immigrant Populations in the New York Metropolitan Area and COVID-19: Lessons Learned in the Epicenter of the Crisis. Acad Med [Internet]. 2020. [cited 2022 Feb 21]. Available from: https://www.ncbi.nlm.nih.gov/labs/pmc/articles/PMC7268828/. 10.1097/ACM.0000000000003518 PMC7268828 32452838

[57] Hayward SE, Deal A, Cheng C, Crawshaw A, Orcutt M, Vandrevala TF (2021). Clinical outcomes and risk factors for COVID-19 among migrant populations in high-income countries: A systematic review. J Migr Health..

[58] Nezafat Maldonado BM, Collins J, Blundell HJ, Singh L (2020). Engaging the vulnerable: A rapid review of public health communication aimed at migrants during the COVID-19 pandemic in Europe. J Migr Health..

[59] Pérez MC, Chandra D, Koné G, Singh R, Ridde V, Sylvestre M-P (2020). Implementation fidelity and acceptability of an intervention to improve vaccination uptake and child health in rural India: a mixed methods evaluation of a pilot cluster randomized controlled trial. Implement Sci Commun..

[60] Jacobson TA, Smith LE, Hirschhorn LR, Huffman MD (2020). Using implementation science to mitigate worsening health inequities in the United States during the COVID-19 pandemic. Int J Equity Health..

[61] Craig P, Di Ruggiero E, Frohlich K, Mykhalovskiy E, White M. Taking account of context in population health intervention research: guidance for producers, users and funders of research [Internet]. 2018 [cited 2021 Aug 17]. Available from: https://www.journalslibrary.nihr.ac.uk/nihr-research/canadian-institutes-of-health-research-cihr-and-nihr-collaboration.htm

[62] Nowell LS, Norris JM, White DE, Moules NJ (2017). Thematic Analysis: Striving to Meet the Trustworthiness Criteria. Int J Qual Methods.

[63] Chen H-Y, Boore JR (2010). Translation and back-translation in qualitative nursing research: methodological review. J Clin Nurs..

[64] Ridde V (2004). Agir contre les inégalités sociales de santé. Can J Public Health..

[65] Ridde V, Guichard A, Houeto D (2007). Les inégalités sociales de santé d’Ottawa à Vancouver: agir pour une « égalité équitable des chances ». Promot Educ..

